# The Role of Influential Actors in Fostering the Polarized COVID-19 Vaccine Discourse on Twitter: Mixed Methods of Machine Learning and Inductive Coding

**DOI:** 10.2196/34231

**Published:** 2022-06-30

**Authors:** Loni Hagen, Ashley Fox, Heather O'Leary, DeAndre Dyson, Kimberly Walker, Cecile A Lengacher, Raquel Hernandez

**Affiliations:** 1 School of Information University of South Florida Tampa, FL United States; 2 Rockefeller College of Public Affairs and Policy University at Albany State University of New York Albany, NY United States; 3 Department of Anthropology University of South Florida St. Petersburg, FL United States; 4 Zimmerman School of Advertising and Mass Communications University of South Florida Tampa, FL United States; 5 College of Nursing University of South Florida Tampa, FL United States; 6 Institute for Clinical and Translational Research Johns Hopkins All Children's Hospital St. Petersburg, FL United States

**Keywords:** COVID-19, vaccine hesitancy, social media, influential actors, influencer, Twitter

## Abstract

**Background:**

Since COVID-19 vaccines became broadly available to the adult population, sharp divergences in uptake have emerged along partisan lines. Researchers have indicated a polarized social media presence contributing to the spread of mis- or disinformation as being responsible for these growing partisan gaps in uptake.

**Objective:**

The major aim of this study was to investigate the role of influential actors in the context of the community structures and discourse related to COVID-19 vaccine conversations on Twitter that emerged prior to the vaccine rollout to the general population and discuss implications for vaccine promotion and policy.

**Methods:**

We collected tweets on COVID-19 between July 1, 2020, and July 31, 2020, a time when attitudes toward the vaccines were forming but before the vaccines were widely available to the public. Using network analysis, we identified different naturally emerging Twitter communities based on their internal information sharing. A PageRank algorithm was used to quantitively measure the level of “influentialness” of Twitter accounts and identifying the “influencers,” followed by coding them into different actor categories. Inductive coding was conducted to describe discourses shared in each of the 7 communities.

**Results:**

Twitter vaccine conversations were highly polarized, with different actors occupying separate “clusters.” The antivaccine cluster was the most densely connected group. Among the 100 most influential actors, medical experts were outnumbered both by partisan actors and by activist vaccine skeptics or conspiracy theorists. Scientists and medical actors were largely absent from the conservative network, and antivaccine sentiment was especially salient among actors on the political right. Conversations related to COVID-19 vaccines were highly polarized along partisan lines, with “trust” in vaccines being manipulated to the political advantage of partisan actors.

**Conclusions:**

These findings are informative for designing improved vaccine information communication strategies to be delivered on social media especially by incorporating influential actors. Although polarization and echo chamber effect are not new in political conversations in social media, it was concerning to observe these in health conversations on COVID-19 vaccines during the vaccine development process.

## Introduction

The rollout of COVID-19 vaccines in the United States has been characterized by high degrees of hesitancy and mistrust. Vaccine hesitancy is deﬁned as “the decision to delay vaccination or the refusal to vaccinate despite available vaccination services” [1]. By mid-2020, only 50% of Americans were estimated to be willing to receive a COVID-19 vaccination right away [2]. Although estimates improved by December 2020, with 70% of Americans indicating they “definitely” or “probably” would vaccinate against COVID-19 [3], hesitancy began to take a sharp partisan turn subsequent to the 2020 election, and uptake has been characterized by acute partisan divides overtaking other forms of hesitancy [3-6]. Nearly 6 months after all Americans aged at least 12 years old became eligible for the vaccine, counties with a larger share of Trump voters had consistently lower vaccination rates contributing to ongoing surges in hospitalizations fueled by the more transmissible Delta variant [6-8]. That vaccine hesitancy should be higher among political conservatives and Trump supporters was not inevitable. Rather, research shows that it may be related to a deliberate strategy undertaken by the antivaccine movement in 2015 to pivot to the far right under the label of “medical freedom” and the formation of political action committees linked to the American Tea Party and its protests against government interference [9]. Moreover, hesitancy was first amplified by the political nature of the vaccine development process, occurring under intense political pressure to reopen the economy and heightened by public concern about the safety and efficacy of emergent COVID-19 vaccines.

Infodemiology is the science of tracing the “distribution and determinants of information in an electronic medium, specifically the Internet, or in a population, with the ultimate aim to inform public health and public policy” [10]. Due to increasing use of social media for health information-seeking [11], it is becoming increasingly important for public health professionals to better engage with social media [12]. Studies have made progress in measuring *information prevalence* by adopting computational methods to track the trends of public discourse and emotions on social media [13-16]. Although social media holds the potential to raise awareness and positive endorsement of vaccines, these conversations are vulnerable to political manipulation and tend to silo users into echo chambers (where beliefs are reinforced by exposure to repeated information associated with individual attitudes inside a closed system) [17].

Even prior to the COVID-19 pandemic, there was evidence that social media vaccine conversations have been targeted by Russian trolls and bots to purposefully manipulate and stoke antivaccine sentiment for political ends [18]. Antivaccine groups are reported to be more active on social media than provaccine accounts [19]. A study of 1344 tweets with the “vaccine” hashtag (#vaccine) between 2010 and 2016 found that antivaccine tweets were 4.13 times more likely to be retweeted than neutral tweets in comparison to 1.58 for provaccine tweets [19]. Evidence from over 100 million Facebook users found that antivaccine communities had the highest growth during the measles outbreak of 2019, dominating the main vaccine conversation with narratives that were targeted at swaying the undecided group toward greater skepticism. Meanwhile, provaccine groups were isolated within their community believing they were “winning” [20].

These findings highlight the outsized role that the most active influencers on social media play in spreading health information. In fact, a recent study found that just 12 influential people on social media were responsible for 73% of the total antivaccine posts on Facebook [21]. Likewise, the most active 25% of US Twitter accounts create 97% of tweets [22].

To devise more practical eHealth communication strategies, it is crucial to investigate the role of influential “actors” and their contribution to the amplification of vaccine information in targeted networks. This study, therefore, sought to identify the most influential actors related to COVID-19 vaccine conversations on Twitter and describe their communication patterns and content during July 2020, a time when attitudes toward the vaccines were forming but before the vaccines were widely available to the public [23].

## Methods

### Research Questions

Our research examined the following research questions pertaining to influential actors and discourse in the polarization of the COVID-19 conversations on Twitter:

Research question (RQ) 1 was “What distinctive communities naturally emerged within the COVID-19 vaccine conversation on Twitter at a time when COVID-19 vaccine hesitancy was at its peak? What does that community structure look like? “

RQ2 was “Who are the most influential actors in this Twitter conversation? What is the role of science and medical experts in the vaccine conversation?”

RQ3 was “What is the level of engagement of retweeting activities in each community?”

RQ4 was “What are the primary topics discussed among the most influential actors within each community?”

### Data Collection

Data were collected in 2 phases. In the first phase, we collected COVID-19–relevant tweets, and in the second phase, we selected vaccine-relevant tweets from the first data set. We initially collected all COVID-19 relevant data on Twitter using the Twitter application programming interface (API) between July 1, 2020, and July 31, 2020, using a query list composed by the University of Southern California [24]. From the collected Twitter data, we further filtered tweets about vaccines that included any of the following keywords: “vaccine,” “antivaxxers,” “antivaccine,” “coronavirusvaccine,” “vaccines,” “CoronavirusVaccine” (Multimedia Appendix 1).

The keyword sets yielded 1,300,828 tweets, which included a total of 751,691 unique Twitter accounts.

### Data Preparation

A node is a Twitter account, which we interchangeably call an actor when we refer to its behavior. We defined an edge as a retweet focusing on information sharing among Twitter accounts [25,26]. When Twitter account A retweets a tweet created by account B, there is a directed edge from A to B. The weight of an edge is the frequency of retweets from A to B. Our data set yielded a total of 617,497 nodes and 910,483 edges, which we sorted by decreasing order based on the weights of edges in order to sample the most active nodes in the discussion network. Gephi version 0.9.2 [27] was used for data analysis and network visualization, which has upper limits on the size of the data it can handle. Initially a total of 100,000 edges (83,098 of the most active nodes) with the highest weights was sampled, which is the approximate maximum volume of data Gephi can handle with the local machine (Ryzen 5800x 8 core 16 thread CPU, 16 GB of DDR4 memory, and dedicated GPU Nvidia 3060ti). This process is sampling the top 11% of the most highly influential Twitter accounts based on the number of retweets. We called this the “first data set,” which was used to answer RQ2, RQ3, and RQ4.

However, since this initial volume of data was still too large for meaningful visualization, highly active nodes were further filtered by selecting nodes with edge weights of 7 or higher within the giant components. This data set with a total of 7382 edges and 1992 nodes was used for the visualization to answer RQ1. We called this the “second data set.”

### Analysis

**Figure 1:**
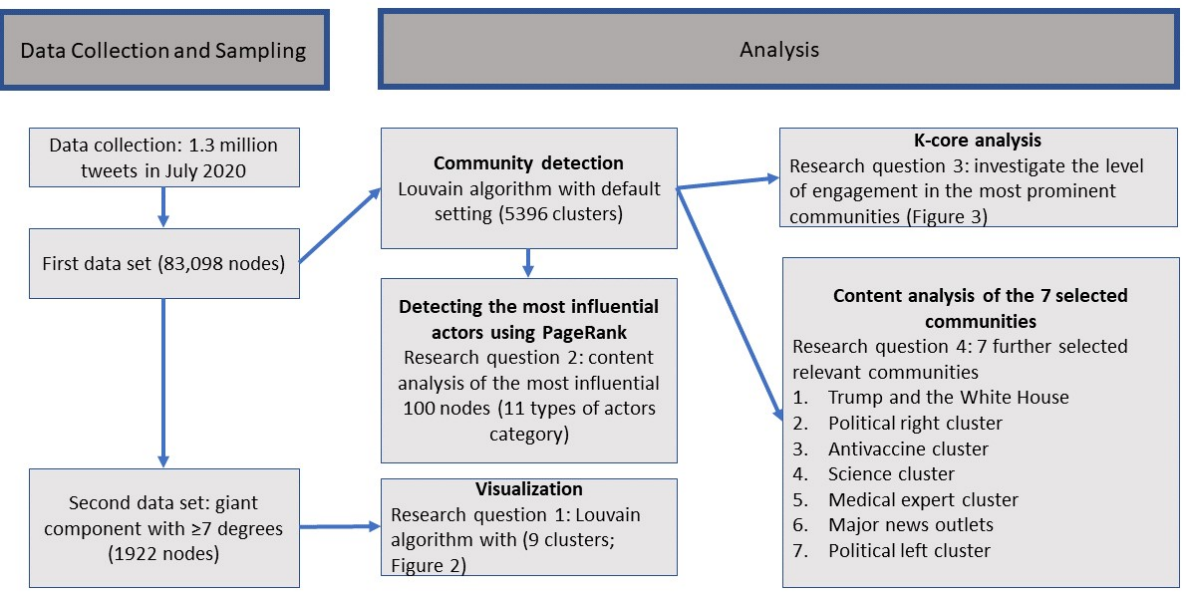
Data collection and analysis.

For rich understanding of a phenomenon from social media data, a mixed methods approach was used by incorporating computational analysis with manual analyses. Figure 1 demonstrates the data collection and analysis process, and the following sections explain the methods used to answer each of the 4 research questions.

#### Community Detection and Visualization

To detect and visualize the social landscape of the communities, Gephi [27], an open source software, was used for network analysis and visualization. To detect naturally emerging communities, the Louvain [28] algorithm, an unsupervised clustering algorithm, was used on account of its high-quality results [29]. The Louvain algorithm automatically creates clusters (or communities) from a given data set by partitioning a network into “communities of densely connected nodes” by separating these nodes from other nodes in different communities [28].

The first data set yielded a total of 5397 clusters using only the default settings of the Louvain algorithm. The top 20 clusters explained about 71% of the nodes, which means that, when using the default settings of Louvain algorithm, many clusters include only a small number of nodes (some contain only 1 or 2 nodes).

We used these initial clustering results, with minimal manipulation, for the sampling data for the annotation tasks in RQ2 and RQ4 and for the K-core analysis to answer RQ3. To make sure that the clustering results were not created from random chance, we ran the algorithm with the same default setting over 10 times and assured that the produced outcomes were consistent—we validated that the network structure was identical and the 100 most influential nodes were almost identical each time.

**Figure 2:**
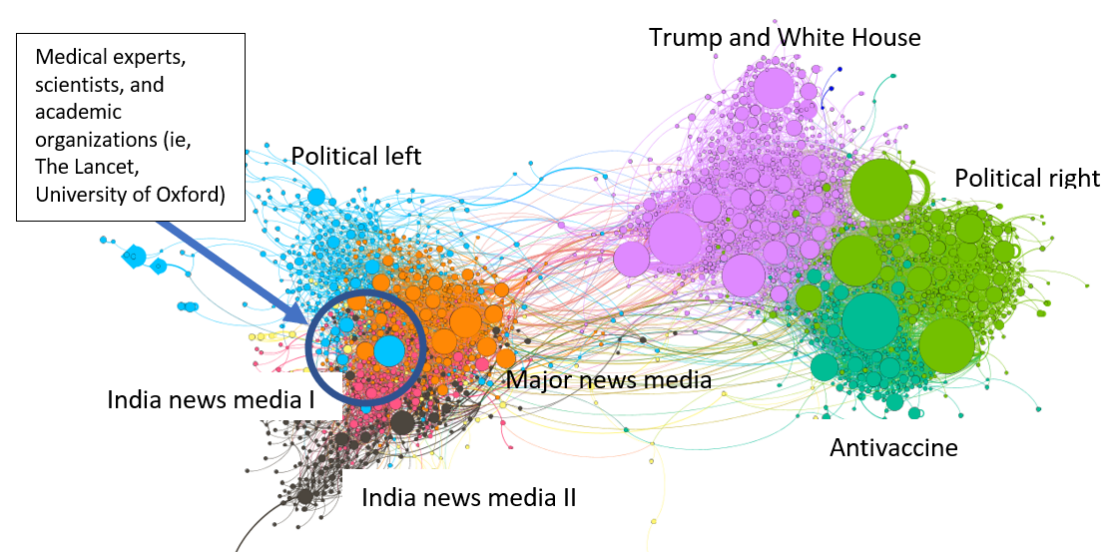
Network graph of Twitter conversations about COVID 19 vaccines using the 1992 accounts with the highest PageRank; 2 clusters (explaining 3% and 0% of all the nodes) were excluded. Node color indicates a unique cluster, and node size indicates the level of influence (according to PageRank), with bigger nodes more influential among the networks.

Since the initial visualization results from the first data set were too complex due to the overwhelming number of clusters, the Louvain algorithm was run one more time using the second data set, which included a smaller number of nodes: those with the most active retweeting behavior (a total of 1992 nodes, ≥7 degree weight). The default parameters of the Louvain algorithm produced 9 clusters with the second data set. Figure 2 is from this second data set. Multimedia Appendix 2 shows the top 7 clusters from the first data set (of the 5397 clusters) and second data set (of the 9 clusters), respectively. All 7 clusters were the same in both results, which validates that the Louvain algorithm produced consistent results with minor proportional changes.

For a spatial representation of the network, we used ForceAtlas2 [30], in which nodes, sharing similar local environments, appear closer to each other. The visualized map shows locations the nodes occupy in networks to indicate the strategic importance of them in specific topic communication.

#### Influential Actors and the Role of Science and Medical Experts in Vaccine Conversations

Diverse measures were available to quantitatively capture the level of influentialness of a node. Betweenness centrality measures may capture the high brokerage potential of a node. Eigenvector centrality can measure the level of popularity of a node based on the connection to other important nodes. PageRank [31] is a variant of eigenvector centrality that counts if a node is endorsed by important nodes. PageRank, formulated by Page and Brin [31], was developed to measure the level of influentialness of a website by giving weights to a website with a higher number of incoming links by *other important* *websites*. We used PageRank to measure the influentialness of a node because a high PageRank value indicates trust and reliability of a node [32], instead of the eigenvector centrality that simply measures the popularity of a node. In our data, a node with a high PageRank means that the node is highly endorsed and trusted by others because its content is frequently retweeted by *other important nodes*.

The goal of this work was to investigate the types of influential actors in the vaccine discussion and to understand the role of scientific and medical experts. In the preparation of the analysis, we sampled the top 100 most influential nodes based on the PageRank value. First, the 30 most influential accounts, according to their PageRank value, were used to develop a category scheme of actor types. To develop the categories of these actors, researchers manually reviewed (1) publicly available Twitter profiles, (2) tweets created or shared by these influential actors, and (3) subsequent web searches (ie, Wikipedia pages) when necessary. A total of 11 actor categories were developed (see Table 1), and 30 accounts were enough to reach a saturation. Second, in order to have a robust categorization of the influential actor types, 70 additional nodes with the highest PageRank value were further sampled. A graduate student followed the category scheme to code the additional nodes. The first researcher revisited the later codes to validate the coding and double checked the consistency. The researcher further consulted with vaccine and medical experts to validate the coding results.

**Table 1:** Category scheme developed for the annotation of actor types.

Number	Category scheme	Definition
1	News media	Mainstream news media
2	Activist	Individual actors, not organizations, who campaign to bring about social and political changes
3	Partisan	An individual or official account in which the main goal is to support a political figure or a political party
4	Medical expert	An individual with an official medical expertise (ie, medical doctor, researcher, and registered nurse)
5	Academic institution	An official account representing academic institution (ie, universities, medical journals)
6	Culture	The main content of the Twitter account is about culture (ie, a BTS fan account)
7	Government	A government organization
8	Business	A company’s official account or an account that clearly pursues financial gain
9	Politician	Elected officials
10	Random individual	A personal account that does not correspond to any of the above categories
11	Suspended	An account that existed during the data collection but was suspended before the category development phase

### Ethics Review

The institutional review board (IRB) of the leading author’s institution responded that our work was considered to be not-human subject research; therefore, IRB review and approval was not required. Although it is not legally required, our research team decided to follow the best practices for ethical Twitter research [33]. The Belmont principle of “respect for persons” requires receiving informed consent from the study subjects. Receiving informed consent from a large data set is not feasible. Instead, Fiesler and Proferes [33] suggested that scholars should identify users only when “the benefits of doing so clearly outweigh the potential harms.” Our goal was to identify the role of the accounts, not the specific identity of the accounts. Revealing the identity of personal accounts may violate the *respect for persons* principle considering the majority of Twitter users are not aware of use of tweets by researchers, and thus feel that researchers should not be able to use tweets without consent [33]. One exception might be “verified accounts” for which Twitter provides a blue badge for accounts “that are of high public interest.” Since this verification process requires the account owners to apply for it *by themselves* and only specific types of accounts are eligible (ie, government, news organizations, activists) [34], we can safely assume that the owners of verified accounts are “public figures” who “waive a substantial part of their right to privacy” for academic research purposes [35,36]. We anonymized personal and unverified Twitter accounts to protect the privacy of these users and only revealed the account names of “verified” accounts.

### K-Core Analysis

K-core analysis was used to investigate the level of tight connections. A k-core is “a maximal group of nodes, all of which are connected to at least K other nodes in the group” [37]. For example, K=3 means that every member of the clique (a small and highly interconnected group) is connected to at least 3 other clique members. K-core, a relaxed measure of a clique, is a measure to capture the level of interconnectivity. *Clique* is a term that refers to a small and highly connected group in which all nodes in the clique are connected to all other nodes. Identifying cliques is important because information can be shared quickly within a clique and members of a clique behave in a cohesive manner [37].

### Inductive Coding

Lastly, inductive coding was conducted by manually reading tweets assigned to each cluster. In preparation for the inductive coding, 7 clusters were purposefully selected from the first data set: 5 of the biggest clusters (political right; major news media; antivaccine; Trump and the White House; political left) were selected (see the size of the clusters in Multimedia Appendix 2), and 2 clusters (science, medical experts) from the top 20 clusters were purposefully included in the sample because we were interested in the role of scientists and medical experts.

This was followed by sampling a maximum of 200 tweets created by the top 5 Twitter accounts with the highest PageRank value from each of the 7 selected clusters. Inductive coding techniques, modeled on grounded theory, were used for the analysis. The first coding phase used open coding followed by a second phase, axial coding, to document trends in each cluster for (1) thematic topic of concern; (2) manifest content such as explicitly stated vaccine risks or benefits and actors (beneficiaries or agents); and (3) latent content such as the function of discourse. The third phase used selective coding to yield brief summaries of patterns in the coded clusters. To establish intercoder agreement in each cluster’s blind coding, 10% of each cluster’s coded data were randomized and verified to exceed 90% agreement. Following practices of social reliability in qualitative research [38], disagreement was discussed and collaboratively recoded as “code unspecified.” If a cluster had more than 10% of codes that disagreed in the sample, the entire cluster was coded by the second coder, and differences were again discussed. This created higher metrics of researcher social reliability [39], which improves the overall accuracy and validity.

## Results

### Naturally Emerging Communities

Using the second data set (a total of 7382 edges and 1992 nodes), the Louvain algorithm automatically detected 9 clusters and assigned numeric values (from 0 to 8) to each of the cluster. Since assigned numbers are not meaningful, we assigned meaningful labels to the biggest 6 clusters (2 clusters were too small to discuss, and 2 clusters were adjacent to each other and thematically the same, so we combined the 2 as Indian news media). The labels were decided based on the user profile description of the top 10 most influential nodes (based on the PageRank value) in each of the clusters.

Figure 2 shows the labels of the 6 major clusters (Trump White House, political right, political left, major news media, Indian news media, and antivaccine). The biggest community was Trump White House (27%), followed by political right (15%) and political left (14%). Figure 2 illustrates that the political right and antivaccine clusters included the most influential actors (the bigger sized nodes are more influential actors in the entire discussion network in Figure 2).

Analyzing the relationship among the clusters, academic organizations and medical experts (discussed in the Primary Topics Discussed Within Communities section) were located close to the major news media and political left clusters (in Figure 2). This means that the major news media and political left tend to depend on information sources from scientific sources and medical experts in contrast with the political right and antivaccine activists who tend to depend on their own information sources.

### Most Influential Accounts and the Role of Science and Medical Experts in Vaccine Conversation

Using an iterative coding approach, a total of 11 categories were developed (academic organization, activist, business, culture, government, medical expert, news media, partisan, personal, politician, and suspended) for the manual coding of the 100 most influential accounts. Table 2 presents the manual coding results, reporting that the news media (27%) and partisan actors (20%) were the biggest actor categories. The polarized network graphs and active involvement by supporters of President Trump showed direct involvement of politics in COVID-19 vaccine discussions. In an inquiry to find the role of science and medical experts, results showed that only 10% and 2% of the 100 most influential accounts were medical experts and academic organizations, respectively. The activists, explaining 11% of the 100 most influential actors, were either antivaccine activists or “conspiracy theorists” who believe that COVID-19 is a human-engineered disaster. Multimedia Appendix 3 shows the account names and the typology of the top 11 partisan actors (in red font). The partisan actors are more frequently from the Trump White House and political right clusters. A total of 5 accounts was suspended, all of which (blue font in Multimedia Appendix 3) appear on the right side of the polarized network. Suspension follows Twitter’s internal policy, whereby accounts are suspended mainly for spamming, security at risk, abusive tweets, or abusive behavior [40].

**Table 2:** Categories of the top 100 influential actors.

Category	Description and verified Twitter handles^a^	Frequency, n
News media	Major news media such as Bloomberg, Reuters, and the Associated Press	27
Partisan	16 accounts out of 20 were Trump supporters. All the verified accounts were @TeamTrump, @ASlavitt, @ksorbs, @charliekirk11, @TrumpWarRoom, @AndrewHClark, @Jillie_Alexis, @AntonioSabatoJr, @tribelaw	20
Activist	7 accounts had antivaccine attitudes; 4 accounts were so called “conspiracy theorists.” Verified accounts were @Jimcorrsays, @RobertKennedyJr	11
Medical expert	@Drdavidsamadi (urologist and Fox News pundit); @FaheemYounus (MD and Chief of Infectious Diseases at a university hospital); @DrEricDing (epidemiologist, National Foundation of Infectious Diseases); @ProfKarolSikora (oncologist)	10
Academic Organizations	*@*UniofOxford, and @TheLancet	2
Others	Government (n=2), business (n=2), culture (n=6), personal (n=10), suspended (n=5), politician (n=5)	30

^a^The coding took place in December 2020. It is possible some account statuses could have changed since our initial coding.

### Level of Engagement in Each Community

**Figure 3:**
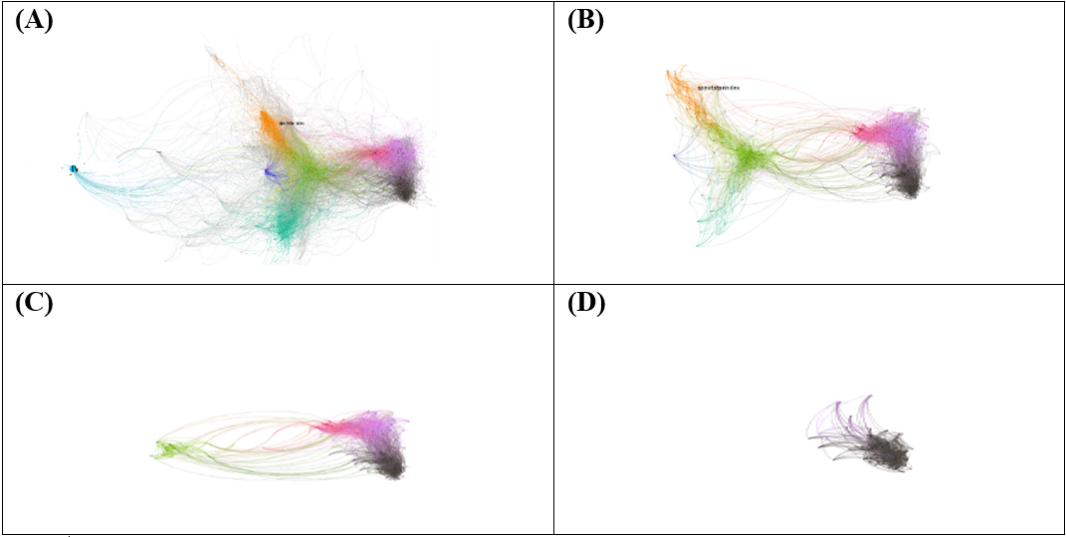
K-core graphs demonstrating the density of groups: (A) 2-core, (B) 5-core, (C) 6-core, and (D) 7-core.

K-core was investigated by eliminating minimal edge connections with other nodes. The 7-core graph in Figure 3 shows that the antivaccination group is a “tier one” group, which includes actors who are densely connected to each other by heavily retweeting content generated among themselves. Many actors in the antivaccine group were connected to at least 7 other clique members, most of whom were in the same community. Therefore, information can be shared quickly within the antivaccine group, and members of this clique behave in a cohesive manner. In contrast, the political left, science, and medical expert communities lost most of the cliques by 5-core. This means that actors in these communities are less cohesive and depend on heterogeneous information sources compared to those in the antivaccine group.

### Primary Topics Discussed Within Communities

In order to understand the content discussed in the major communities, we conducted content analysis of tweets created and shared by influential actors. The result showed the concerns of the political right and antivaccine community included vaccine safety and infringing on rights and liberty. The Trump and White House and political left clusters used vaccines as a partisan tool to gain political advantage. The following paragraphs provide summaries of the inductive coding results for the 7 selected clusters.

The content of the Trump and the White House cluster showed the COVID-19 vaccine was a major presidential deliverable as evidence of political legitimacy in the present and future. This cluster’s tweets were highly partisan, showing President Trump was successfully managing the rollout of the vaccine—hence worthy of political trust (eg, White House Press Secretary @KayleighMcEnany: “These critical investments in a coronavirus vaccine are due to the fact that we have a businessman in the White House.” [41]).

Two major arguments that were repeatedly called out in the Tweets were (1) use of the COVID-19 vaccine to demonstrate Trump's sound management and growth of the national economy, legitimizing his presidency, and (2) use of “speed of planning” for vaccine development, manufacturing, and distribution to demonstrate Trump's capable management of complex national processes, again legitimizing his presidency. These markers—money and time—were likewise used in some oppositional Tweets proposing political mismanagement by Biden.

The content of the political right cluster was closely linked to themes of antivaccine and Trump and the White House clusters, with about one-third citing conspiracies, often naming the untrustworthy beneficiaries with motives of depopulation, corruption, and DNA disruption: “Bill Gates vaccine agenda #DEPOPULATION Fauci awarded a $3.7M research grant to the Wuhan lab, working on BioW Coronavirus. Wuhan labs wants to Patent Gilead’s Remdesivir. Fauci is on the board of Gates Foundation. Gates gave CDC $13.5M & is second largest funder to the W.H.O.*”* [42].

This cluster also expressed distrust of Big Pharma, the Centers for Disease Control and Prevention (CDC) and scientific and medical decision makers. Surprisingly, the cluster indicated a degree of distrust in Big Government and the Trump administration; when present, the distrust was largely related to suspicion of direct financial benefits (patents, stock ownership).

Other conspiracy themes included unauthorized collection of personal information (eg, DNA, tracking). An emergent conspiracy theme in the conservative activist cluster included beliefs that the vaccine was deliberately designed to depopulate through killing recipients or causing sterility. This cluster also made accusations tying them as conscious agendas of the social movement and having a reluctance to mandatory vaccination challenging their American values of freedom and liberty. Conspiracies, paralleled in less radical tweets, reported government and pharmaceutical sectors negotiated release from liability for known side effects from the vaccine. Extreme conspiracies cited cover-ups for massive death and complication rates in ongoing human trials that were complemented by vaccines being unnecessary due to supposedly promising alternative treatments and therapies—most notably hydroxychloroquine.

The antivaccine cluster was highly connected within and with the political right cluster. The top 5 influential nodes in the antivaccine cluster were either not verified (n=4) or suspended (n=1). Topical trends related to conservative ideology including freedom and rights, and the forcible control over citizen’s actions and bodies included narratives like the arguments in the political right cluster. Topical trends included that the vaccine was unnecessary or ineffective, referencing claims of health, fitness, and cognitive ability to beat an infection. The topic of lack of trust of Big Pharma and the justice system was expressed as suspicion of releasing manufacturer liability by minimizing vaccination risks and manufacturer culpability. Mistrust was high in specific conspiracies in this cluster that often linked Big Tech, Big Pharma, and Big Government. Topics also included DNA disruption and fears of adding unknown substances to the vaccine like tracking devices, genetic material theft, and general “unknown” materials. There were few scientific claims; more widespread were claims of censorship.

The content of the science cluster indicated broad approval and encouragement of the vaccine and its makers with some distrust against the Trump administration. The latter explains the distance between the Trump and the White House cluster and scientists in Figure 2. Expressed concerns centered around the rapidity of vaccine development with compromise of safety. This content was saliently tied to Trump in 3 core ways: (1) Tweets criticized Trump’s rapid vaccine rollout citing it as the “October election vaccine;” (2) tweets focused on corruption and equity, demanding Trump provide a universal, free vaccine; and (3) tweets highlighted Trump’s retweets of a doctor who claimed the vaccine was from alien DNA, reinforcing his schism with the scientific community and principles. This cluster did demonstrate some trust of the Trump administration through the surrogate of Fauci as expressed in tweets on Fauci’s role and advice being “spot on” or discussed his disinterest in the vaccine “race” as against Russia, all highlighting partisan messaging during the development process. Aside from these fears, this cluster exhibited apprehensions about the growing role of antivaccine advocacy groups with general concerns that “antisocial” messages will coincide with the public inoculation timeline.

The content of the medical expert cluster demonstrated similar themes to the science cluster by containing specific evidence—for example, not just documenting progress milestones but including supporting descriptive statistics. Like the media cluster, references and links were used to promote longer content with articles and interviews serving as evidence. This cluster used historical and comparative rhetoric with other diseases and responses, but unlike other clusters, used the historical or comparative references to overcome current barriers or cause for optimism. Similar to other clusters, structural limitations in manufacturing and distribution were raised. A functional clue to expected audience is demonstrated in the dissemination of medical analyses and science claims, noted by dense jargon without a primer for the public. Although a small number of tweets actively engaged the malignment of the vaccine by addressing antivaccine propaganda or misinformation, it was not distributed among many accounts. Likewise, conspiracy tweets from (2) accounts demonstrated how people without medical expertise cross talked the medical cluster.

The content of the major news media cluster involved broad support for the vaccine and its makers and differed from other clusters in its analysis of the vaccine narrative through 3 main topics. First, nearly one-quarter of the content either documented the status of vaccine manufacturing progress or suspected date of availability. Second, a timing theme speculated about plans for vaccine manufacture and distribution. Although other clusters were concerned about timing, the media’s concern largely focused on milestones and speculated about deliverables, rather than expounding on vaccine safety risks or economic outcomes. Third, elements of skepticism and distrust were present in the form of the attention given to “dose deals” (where countries contracted future access to vaccines) and ethical violations (intellectual property violations, deliberate risks to human trial volunteers). This was complemented by content with overt and secondary implications of vaccine nationalism or of international cooperation. Although political implications were present in many tweets, they were less partisan in nature than other clusters.

The political left cluster contained mixed messages about trust regarding the vaccine. A small number believed the vaccine is one method to combat the virus, most were divisive, and approximately 20% circulated conspiracy theories. Contributors expressed distrust through vaccine hesitancy patterns; others expressed trust that the vaccine is the “lynchpin” by which society can return to normal. Within this cluster, there were claims that (1) vaccine science is sound, but Trump’s political manipulation of the timeline to optimize the election has compromised the trusted process; (2) vaccine manufacturing is being used as a conspiratorial economic investment to Trump’s allies, again compromising the manufacture and distribution; and (3) the Trump administration was subverting American ethics like hard work, integrity, and innovation by ignoring or supporting international violations of intellectual property. This cluster also included a pattern of partisan rhetoric in tweets that explicitly used Trump as a metaphor or symbol for the virus (eg, the Trump Virus or Trump is the Virus/Biden is the Cure).

## Discussion

### Principal Findings

Using network analysis and unsupervised machine learning with samples from Twitter data and conducting inductive coding to characterize tweet discourse, we found that, during this period, COVID-19 vaccine Twitter conversations were already highly polarized. The most influential Twitter actors were not scientists and medical experts but rather partisan actors and antivaxxers. Actors on both the political left and political right expressed skepticism and misgivings toward the COVID-19 vaccine development process but were motivated by different concerns and used different language to describe their concerns. Conspiracy theories were raised on both sides.

Our analyses of Twitter posts during the height of stay-at-home measures in the United States and amid the race to develop COVID-19 vaccines demonstrated a high degree of Twitter social media activity related to vaccine development. Twitter vaccine conversations were highly polarized, with different actors occupying separate “clusters,” reinforcing concerns about “information bubbles.” The level of polarization was similar to a deeply political event such as the Muller investigation of Russian interference in the 2016 US elections [43].

Media and science or medical actors were especially absent from the conservative clusters, and antivaccine sentiment was especially salient in the political right cluster. Results also showed “antivaccine” groups to be highly engaged actors in the COVID-19 vaccine conversation, circulating information particularly within a tight conservative cluster.

### Health Information Sources and the Politicization of Science

These findings have important implications for health professionals’ communication and education about vaccines. Although it may not be public health professionals’ traditional roles to address the politicized nature of vaccine acceptance, it is increasingly important for them to understand how patients gather health information from online platforms and “adjudicate the merits of such information” [11].

Previous research has shown how a small but influential handful of actors with medical credentials or authority can disproportionately sway Twitter conversations and promote the spread of misinformation. This misinformation can then be further amplified by partisan actors who misrepresent and exaggerate these statements for political gain. For instance, Haupt et al [44] found that a single group claiming medical and scientific credibility and authority (ie, Dr Immanuel and America’s Frontline Doctors) successfully promoted the use of hydroxychloroquine, even though the efficacy of hydroxychloroquine had not yet been fully demonstrated. Political (eg, Trump) and media sources then amplified and disseminated this information in support of the use of hydroxychloroquine [44]. Our empirical evidence shows that the vaccine conversation had already become politicized along partisan lines before the vaccine was available, with vaccine acceptance driven by ideological beliefs and attitudes, particularly among Twitter influencers. As previous research shows, when disease threats become partisan, or “politicized,” people look to their preferred political party to decide how much they ought to worry [45,46]. Once politicized, issues can be hard to depoliticize, and rather than looking to science or medical experts, people look to less credible sources of information or adopt practices, such as vaccine refusal, that may be hard to alter.

The politicization of the COVID-19 vaccine conversation is an important empirical outcome because, although politicization of science has existed in environmental politics and policy [47], this is a relatively newer development for public health policy. Although certain public health issues have long been politicized (eg, sexual and reproductive health, HIV policy) [48], other infectious disease threats have not been politicized in the same way as COVID-19. For instance, the findings from this study contrast with the findings from a Twitter analysis during the Zika pandemic. Research showed that the Twitter conversation about Zika was not polarized; instead, there was higher trust in medical and scientific authorities, even though the Zika health crisis data were collected during the summer of the Presidential 2016 election campaign, just as our data set was also collected (July 2020) [49]. By contrast, the issue environment surrounding our data collection was highly politicized during—and even before—the pandemic [45,50,51], thereby enabling partisan actors and political elites, not medical experts or scientists, to play an important role in leading the discourse.

### Political Attitudes, Health Beliefs, and Behaviors

Our findings contribute to knowledge development examining the relationship between political ideology and attitudes toward science [52-54]. Although the conservative network was marked by an overrepresentation of partisan and vaccine skeptic actors and an underrepresentation of science or medical experts, this may be the result of deliberate targeting of political conservatives rather than a reflection of an inherent skepticism of the scientific community rooted in ideological differences [53]. Rather, this research demonstrates that actors on both the political left and political right expressed skepticism and misgivings toward the COVID-19 vaccine but were motivated by different concerns and language, suggesting both conservative and liberal actors are susceptible to political manipulation and framing of issues. Notably, conspiracy theories were present in both liberal and conservative clusters, further supporting the contextual hypothesis that both liberals and conservatives are likely to doubt science if scientific information contradicts their preconceived worldviews [52,54].

Although the analysis of tweets in each cluster revealed highly divided vaccine discourses among specific political communities, “distrust” arose as a common (and primary) construct advanced by partisan actors throughout the content analysis. On the liberal side, general distrust of the COVID-19 vaccine development process was expressed through fears that its speed would compromise safety (science cluster), dosing deals and ethical violations (major news media cluster), and the intentional abuse of the progress timeline for political gain (political left cluster).

Within the political right and antivaccine clusters, themes that emerged included distrust and antivaccine rationales rooted in conservative ideology including language of freedom or rights, forcible control over citizen actions and bodies, and large-scale economic profit.

Evidence that “antivaccine actors” were more heavily present in conservative networks was prominent in this study, and conspiracy theories and conservative ideologies of freedom and rights were prevalent themes that were not previously evident in the vaccine hesitancy literature. This is consistent with concerns that have been raised about how the antivax movement is specifically targeting political conservatives and the far right through a campaign of “medical freedom” to advance their cause [9,55]. Further investigation of this finding may help explain why White Republicans have been identified as the most vaccine hesitant group in recent polls [56,57].

### Implications for Public Health Communication Using Social Media

Given the growing proportion of the population that attains health news through social media [58,59], it is important for public health professionals to harness the power of social media to support situational awareness (ie, public’s behavior, emotion, information demand) [12]. Additionally, the findings from our study and related studies can be used to help identify and counter the narrow group of influencers that are most responsible for amplifying antivaccine sentiment such as through better enforcement of platforms’ existing standards [21].

### Methodological Implications and Limitations

Methodologically, this study demonstrated how to identify the most influential actors during an acute public health crisis and how information clusters have formed on social media at a critical moment when people’s attitudes toward the vaccines were being formed. This knowledge is beneficial for developing health communication strategies on which social media “influencers” to target for information distribution or for counter-messaging.

Nevertheless, there are limitations to this study. First, interpretation of the results of our study are necessarily bounded by the Twitter platform, which does not always reflect the general public (ie, Twitters are younger, likely to be liberal with higher incomes than US adults, and strongly influenced by a small number of prolific users) [60]. Consequently, Twitter reactions do not always reflect overall public opinion [61]. Therefore, the results of this analysis should not be regarded as emblematic of broader attitudes and beliefs on COVID-19 vaccines. Also, we do not know if the platform specifically affected the differences between Twitter and survey studies on vaccine hesitancy discourse. Therefore, future studies may investigate the same phenomenon using different methods. Traditional survey methods rely on self-report and may create incentives for participants to give politically correct responses; however. these do not allow elaboration due to standardized question wordings [62]. By contrast, Twitter data include expression of users in their natural environment and enable synchronous data collection as one’s expressions occur. Therefore, Twitter (or other social media) data include more current sentiments on vaccine hesitancy.

Second, we decided not to delete bots because (1) determining bot accounts requires further investigation on setting a proper threshold, (2) accounts with higher bot scores do not seem to seriously interfere with discussions, and (3) deleting bots means artificially manipulating a raw data set because bots are part of the Twitter ecosystem. Using our data set, we detected bots to investigate to what extent bots are interfering with vaccine conversations by running one of the most popular bot detection algorithms called Botometer [63]. Emulating the study by Hagen et al [43] that found political bots’ effect on the network structure, we investigated the proportions of bots among the most influential actors. We were not able to find outstanding evidence of bots systematically interfering with the conversation in our data set to the extent to justify deletion of bot accounts. More importantly, when we initially set a relatively conservative threshold of a 0.7 complete automation probability (CAP) score following previous Botometer research [64], human accounts were frequently tagged as bots (ie, the Twitter account of former President Obama had a bot score of 0.8). This means that, without further detailed study to decide a proper threshold for the Botometer to accurately detect bots (we conducted a separate study for this), it is better not to delete bots from the data to preserve the natural ecosystem of Twitter.

### Conclusions

COVID-19 vaccine conversations in July 2020 were highly polarized along partisan political lines. Specifically, “actors” on the political right of the spectrum formed a tight information-sharing cluster that was highly siloed and infiltrated by the antivaccine community; this group tended to circulate conspiracy theories and were far less likely to distribute vaccine knowledge from scientific and medical expert clusters. Concerningly, “trust” in a COVID-19 vaccine was highly manipulated by partisan actors on both the left and the right for political advantage. These findings are informative for designing improved vaccine information communication strategies to be delivered on social media. Although polarization and the echo chamber effect are not new in political conversations on social media, it was a concern to observe these in health conversations on COVID-19 vaccines during the vaccine development process.

## Appendix

Multimedia Appendix 1 Full keyword list for data collection.

Multimedia Appendix 2 Supplemental table.

Multimedia Appendix 3 Supplemental figure.

## Abbreviations

API: application programming interface
CAP: complete automation probability
CDC: Centers for Disease Control and Prevention
IRB: institutional review board
RQ: research question
