# Supplementary material for: The Role of Influential Actors in Fostering the Polarized COVID-19 Vaccine Discourse on Twitter: Mixed Methods of Machine Learning and Inductive Coding
Source: JMIR Infodemiology. 2022 Jun 30;2(1):e34231. doi: 10.2196/34231 (PMC9254747; doi:10.2196/34231)
Supplement: Multimedia Appendix 1 [file infodemiology_v2i1e34231_app1.docx]

# Full keyword list for data collection

Coronavirus

Koronavirus

Corona

CDC

Wuhancoronavirus

Wuhanlockdown

Ncov

Wuhan

N95

Kungflu

Epidemic

outbreak

Sinophobia

China

covid-19

corona virus

covid

covid19

sars-cov-2

COVID-19

COVD

pandemic

coronapocalypse

canceleverything

Coronials

SocialDistancingNow

Social Distancing

SocialDistancing

panicbuy

panic buy

panicbuying

panic buying

14DayQuarantine

DuringMy14DayQuarantine

panic shop

panic shopping

panicshop

InMyQuarantineSurvivalKit

panic-buy

panic-shop

coronakindness

quarantinelife

chinese virus

chinesevirus

stayhomechallenge

stay home challenge

sflockdown

DontBeASpreader

lockdown

lock down

shelteringinplace

sheltering in place

staysafestayhome

stay safe stay home

trumppandemic

trump pandemic

flattenthecurve

flatten the curve

china virus

chinavirus

quarentinelife

PPEshortage

saferathome

stayathome

stay at home

stay home

stayhome

GetMePPE

covidiot

epitwitter

pandemie

virus

kung flu

covididiot

wearamask

wear a mask

vaccine

mybodymychoice

righttochoose

vaççine

provax

vaxwithme

vaccine

antivaxxers

antivaccine

coronavirusvaccine

vaccines

CoronavirusVaccine
