# Supplementary material for: The Role of Influential Actors in Fostering the Polarized COVID-19 Vaccine Discourse on Twitter: Mixed Methods of Machine Learning and Inductive Coding
Source: JMIR Infodemiology. 2022 Jun 30;2(1):e34231. doi: 10.2196/34231 (PMC9254747; doi:10.2196/34231)
Supplement: Multimedia Appendix 2 [file infodemiology_v2i1e34231_app2.docx]

**Supplement Table 1.** Distribution of the biggest clusters from the first and second datasets

| Label | Freq. (Percent) from the First Dataset* | Freq. (Percent) from the Second Dataset* |
| --- | --- | --- |
| Political Right | 7589 (20%) | 289 (15%) |
| Major News Media | 6420 (17%) | 235 (12%) |
| Indian News Media | 5859 (16%) | 243 (13%) |
| Anti-vaccine | 5728 (15%) | 170 (9%) |
| Indian News Media | 4250 (11%) | 182 (9%) |
| **Trump White House** | 3920 (10%) | 543 (28%) |
| Political Left | 3780 (10%) | 271 (14%) |
| Total | 37,546 (100%) | 1,933 (100%) |

*We present the top 7 of a total of 5397 clusters from the first dataset, and the top 7 of a total of 9 clusters from the second dataset. The biggest 7 clusters (out of 5397 clusters) explain 45% of the first dataset, and the biggest 7 clusters (out of the 9 clusters) explain 97% of all the second dataset. Although the Political Right was the biggest cluster from the first data set, the Trump White House cluster was the biggest in the second data set. This means that selecting the most active accounts (based on the edge weight did not change the biggest clusters, which validates that selecting the biggest clusters did not bring any changes in terms of the major clusters. Within the 7 clusters, there are some changes in the proportion of the size.
