# Supplementary material for: The Role of Influential Actors in Fostering the Polarized COVID-19 Vaccine Discourse on Twitter: Mixed Methods of Machine Learning and Inductive Coding
Source: JMIR Infodemiology. 2022 Jun 30;2(1):e34231. doi: 10.2196/34231 (PMC9254747; doi:10.2196/34231)
Supplement: Multimedia Appendix 3 [file infodemiology_v2i1e34231_app3.docx]

**Supplement Figure 1.** Network graph of Twitter conversation about COVID 19 Vaccine that includes the most influential actors from Medical Experts, Science, and Major News Media


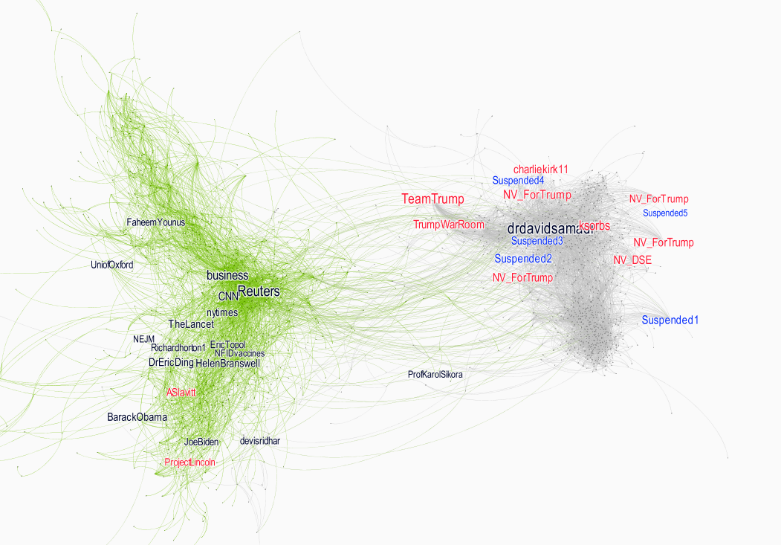


**Notes**: Red fonts are partisan actors. Blue fonts are suspended accounts by Twitter. Black fonts include influential actors from News Media and Medical Expert categories. Compared to Figure 1, the Indian News Media clusters and the Political Left clusters are transposed in this version due to the unsupervised nature of the clustering algorithm.

When an account was not verified or not a public account (eg, government or university accounts), the account name was anonymized and was labeled NV_.
